# Psychological changes and associated factors among patients with tuberculosis who received directly observed treatment short-course in metropolitan areas of Japan: quantitative and qualitative perspectives

**DOI:** 10.1186/s12889-019-8001-9

**Published:** 2019-12-05

**Authors:** Kae Nagahiro Shiratani

**Affiliations:** Graduate School of Medicine, Yokohama City University, 3–9 Fukuura, Kanazawa-ku, Yokohama, Japan

**Keywords:** Tuberculosis, Post-traumatic growth, Directly observed treatment short-course, Patient-centered care, Recovery, Public health nurse, Japan

## Abstract

**Background:**

The directly observed treatment short-course (DOTS) is one of the most effective tuberculosis (TB) control measures worldwide. However, despite its aim of providing comprehensive and humanistic care, few studies have examined its psychological effects from the patient’s perspective. Thus, this study aimed to evaluate the psychological changes and identify associated factors among patients with TB undergoing the DOTS program in Japan.

**Methods:**

This cross-sectional study recruited patients with TB receiving the DOTS program via 32 public health centers in four metropolitan cities in Japan. Surveys were administered to the patients and their attending public health or clinical nurses, who were responsible for their care and the DOTS program. Data were collected regarding the patients’ demographic and clinical characteristics, post-traumatic growth (using the Post-Traumatic Growth Inventory-Short Form [PTGI-SF]), and medication adherence, alongside open-ended questions, from 2014 to 2015. Additionally, the patients’ appraisal of the DOTS program’s efficacy and nurses’ assessment of the program’s practices were measured using two original questionnaires. Factors associated with post-traumatic growth were analyzed using variable estimation, correlation analysis, and logistic regression. Thematic analysis was conducted on the open-ended responses.

**Results:**

Questionnaires were returned by 127 patients (125 valid answers); 98 (78.4%) of the respondents were men. Their mean age was 63.3 (standard deviation: 15.8) years. The mean PTGI-SF score was 21.7 (standard deviation: 11.1). The logistic regression analysis found that post-traumatic growth was significantly associated with the patients’ appraisal of the program’s efficacy (odds ratio [OR] = 1.157, 95% confidence interval [CI] = 1.026–1.304) and nurses’ assessment of the practices (OR = 1.307, 95% CI = 1.065–1.603). In the qualitative analysis, “Non-acceptance,” “Frustration,” and “Anxiety” were extracted as barriers to treatment; “Fear,” “Acquiring a partner,” “Relief,” and “Belief” were extracted as treatment drivers; and “Life changes” and “Rebuilding oneself” were extracted as treatment outcomes.

**Conclusions:**

The DOTS program in Japan improves patients’ treatment adherence and leads to recovery and psychological growth. Even in other regions, it may be effective to incorporate this program’s practices that place importance on partnerships with patients. It is also necessary to continue refined quantitative and qualitative evaluations.

## Background

Tuberculosis (TB) has one of the highest global mortality burdens of any disease, and while its overall incidence is on the decline, the threat to the developing world is still serious [1]. In the developed world, TB prevalence is decreasing due to socioeconomic progress and improvements in patient detection and treatment outcomes. However, this progress has been stymied by population inflows from TB-endemic areas and high infection rates among the urban poor, and, as in the rest of the world, recent surges in cases of multidrug-resistant TB and human immunodeficiency virus co-infections are serious issues [1]. Japan still experiences more than 17,000 new TB cases per year [2]. Moreover, due to mass infection among construction workers, the homeless, and so on, frequent outbreaks have occurred among the socially and economically disadvantaged in cities [3], and other issues have created an abundance of complex and intractable cases. Researchers and policymakers alike are exploring ways to treat and cure TB in these high-risk populations.

The directly observed treatment short-course (DOTS) has been advocated by the World Health Organization as one of the most effective TB control measures and it is implemented as standard in many countries [4–6]. In practice, according to the WHO’s DOTS strategy, the contents and specifics vary between countries and even regions [7]. Japan began its own national-level DOTS program that targets public health centers across the country (see the detailed description in the Methods section) [8].

How effective is DOTS? A Cochrane systematic review found that DOTS-based strategies were not significantly different for resolving poor adherence to TB treatment than self-administered therapy considering the large amount of resources required and the cost implications, and the authors recommended motivating patients and DOTS staff [9]. Thus, how can we improve the poor adherence or motivate the high-risk population? The efficacy of a given DOTS strategy must first confirm their understanding of the intervention.

Another concern is the strong tendency for DOTS-related research to discuss such programs from a purely medical perspective and to consider their efficacy mainly in terms of treatment outcomes and patient medication status [10]. Qualitative research has shown that other aspects, including psychological factors, are an important component of the efficacy of DOTS [11]. For example, Nagahiro [12] examined how patients with TB experienced treatment with the DOTS program in Japan, and it was found that, in this program, they tried to cherish their respect and care for their personal needs as they realized the meaning and worth of their lives. Kawatsu [13] showed that the DOTS program in Japan worked both to control TB and to empower patients by addressing their emotional needs. The association between TB and mental health issues, including symptoms of post-traumatic stress disorder [14], emphasizes the importance of evaluating its psychosocial dimensions.

Despite the strategy’s stated aim of providing comprehensive and humanistic care, few studies have examined the psychological effects of the DOTS program, including in terms of post-traumatic growth, which is a construct defined as the positive psychological change resulting from the struggle with a highly challenging, stressful, and traumatic event [15]. Therefore, this study aimed to identify the factors associated with post-traumatic growth among patients with TB who were involved in the DOTS program in Japan, and it examined these factors using qualitative analysis to investigate the experiences of these patients.

## Methods

### Conceptual framework

**Fig. 1:**
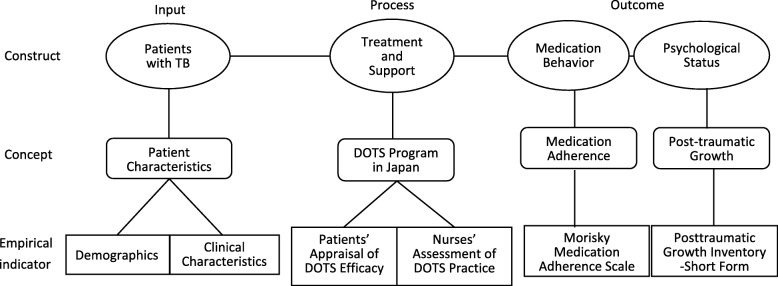
Conceptual model of this study

Based on a previous study [16], the conceptual model of the current quantitative study was constructed (Fig. 1) to determine the different constructs and concepts pertaining to the lives of patients with TB undergoing the DOTS program in Japan and to examine the respective evaluation tools.

The input included the patients undergoing the DOTS program, which was measured in terms of their demographic and clinical characteristics. The processes included in the DOTS program in Japan were measured using two indicators: the patients’ appraisal of the efficacy of the DOTS program and the nurses’ assessment of the DOTS program’s practices*.*

**Fig. 2:**
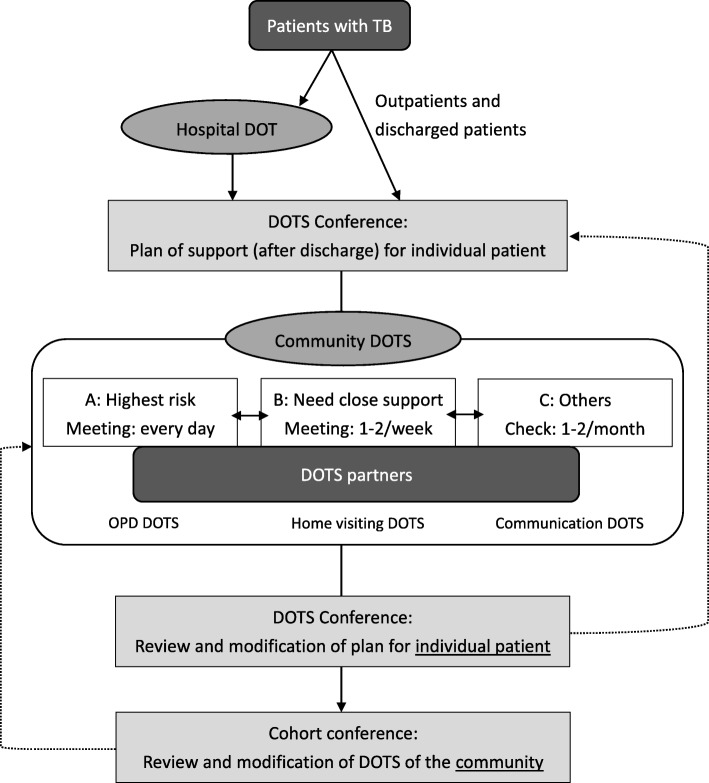
The DOTS program in Japan

The DOTS program in Japan is presented in Fig. 2 [8]. This program can be understood as a comprehensive medication and treatment support system, which supplements the WHO’s DOTS strategy with consultation and educational elements. It consists of several components, including “Hospital DOTS,” which is led by medical institutions, and “Community DOTS,” which is led by public health centers in collaboration with related organizations. These efforts are supplemented by DOTS conferences to customize support plans based on patients’ individual circumstances and evaluation meetings to assess the treatment outcomes and DOTS approaches. Community DOTS targets discharged patients and patients receiving outpatient medical treatment, as well as newly released ones. Enrollees are classified into one of three types (A, B, C) based on an assessment of their non-adherence risk and provided with a corresponding level of support. Directly ensuring that Type A patients—the category at highest risk of discontinuation—take their medication every day is an integral component of their care; they also may receive outpatient DOTS at medical clinics and home-visit DOTS by public health nurses. Type B patients also need close medication and treatment support: once or twice a week, they must take their medication under the direct observation of public health or home visiting nurses or pharmacists (pharmacy DOTS). Type C covers all remaining cases that need support communication: these patients undergo DOTS by means of interviews, phone calls, or letters once or twice a month. In addition, all patients are given good explanations of their disease and treatment and the suitable environments for taking their medication, and are provided with mental health support. Further, this program has been supported by DOTS meetings for examining individual support plans and cohort meetings for reviewing DOTS in the community.

The outcome of the conceptual framework was related to the concept of medication adherence and the concept of post-traumatic growth of the patients with TB receiving the DOTS program in Japan.

### Design

This study employed a cross-sectional design. Questionnaire surveys that included both quantitative and qualitative (open-ended) items were distributed to patients with TB and their attending nurses in four major cities across Japan—Yokohama, Kawasaki, Nagoya, and Osaka—with an estimated total population of 10 million in 2015 [17]. The reason for conducting both quantitative and qualitative examinations is to identify the factors related to the lifestyle and psychological transformations among individuals undergoing treatment for TB as well as to explore the actual perceptions, multi-layered viewpoints, and cultural influences of patients in the DOTS program [18].

### Sample size and participants

The sample size was calculated using G*Power v. 3.0.1, with 80% power, *p* < 0.05 as the statistical significance, an effect size of 0.2, and eight predictors. The estimated minimum sample size required to identify the factors associated with post-traumatic growth was *n* = 75 [19]. With an expected response rate of 20%, the number of expected questionnaires was 375.

Participants were recruited from 32 public health centers or their branch offices in the four cities stated above using purposive sampling; they were referred by the public health nurses or clinical nurses in charge of their care. The inclusion criteria were as follows: a diagnosis of TB (including TB pleurisy), participation in the DOTS program, and receipt of care from public health or clinical nurses for more than 1 month. Altogether, 375 questionnaires were distributed to dozens of public health nurses between March 2014 and February 2015. The areas mentioned above were selected because they all fall within the top seven municipalities in Japan in terms of TB morbidity [20]. Moreover, within these cities, some areas have an extraordinarily high TB prevalence, which indicates that they face the greatest health risks. These factors ensure that any conclusions drawn about the implementation of the DOTS program’s practices in these areas are highly relevant and significant for public health.

### Measures

The primary outcome variable in this study was post-traumatic growth [15]. This construct was measured using the Japanese edition of the Post-Traumatic Growth Inventory-Short Form [PTGI-SF] [21], which consists of 10 items pertaining to different types of positive change. Participants rate each item on a six-point scale ranging from 0 (no change) to 5 (a great degree of change). Total scores range between 0 and 50, with higher scores reflecting greater growth [22]. The validity of the Japanese edition of the PTGI-SF has been confirmed through exploratory and confirmatory factor analyses (NFI = 0.938, CFI = 0.955, TLI = 0.94, RMSEA = 0.088), and its internal consistency has been demonstrated (overall item reliability coefficient: 0.90) [21].

The independent variables included self-reported sociodemographic information (e.g., age, sex, type of residence, household, occupational status, and educational level) and clinical characteristics (e.g., TB symptom severity, ability to move at the time of the diagnosis, comorbid diseases, and understanding of medication guidance). TB symptom severity was evaluated by a question with the following options: “I barely had any symptoms” (coded as mild), “My symptoms were painful, but did not interfere with my lifestyle” (moderate), and “My symptoms were so painful they interfered with my lifestyle” (severe). Medication adherence was assessed using the Morisky Medication Adherence Scale [MMAS-4]: participants were considered non-adherent if they responded “true” to any of the four questions [23]. Recovery status by treatment was evaluated with the following question: “Do you feel better/unchanged/worse since the treatment started?”

The patients’ appraisals of the efficacy of the DOTS program and nurses’ assessments of the DOTS program’s practices were measured using original questionnaires based on a literature review, interviews with 26 patients [12], and interviews with eight public health nurses [16]. The Patients’ Appraisals of the DOTS Program Efficacy [24] were assessed using nine questions, such as “Do you feel supported in taking your medication by the collaboration between different sections, such as public health nurses, registered nurses, pharmacists, physicians, and home helpers?” A three-factor structure was extracted via item and factor analysis of 116 respondents (Cronbach’s α = 0.84), which is presented in Additional file 1. The Nurses’ Assessment of the DOTS Program Practice [24] was assessed by rating the nurses’ responses to nine questions, such as “Do you adjust the necessary stakeholders or sections and resources for your patient so that he/she can continue to be treated?” A three-factor structure was also extracted via item and factor analysis of 88 respondents, (Cronbach’s α = 0.73), which is shown in Additional file 2. In both questionnaires, participants rated each item on a five-point scale ranging from 0 (not applicable) to 4 (highly applicable). The total scores ranged between 0 and 36, with higher scores reflecting greater efficacy/practice. The survey also contained a risk assessment component, in which nurses indicated factors that impeded their effective provision of the DOTS program.

The patient questionnaire also included the following three open-ended questions: “What kind of things did you find troublesome as you went through the treatment?” “What kind of things did you find helpful as you went through the treatment?” and “How did your feelings, thoughts, behaviors, and lifestyle change after starting the treatment?”

## Analysis

### Statistical analysis

Descriptive statistics were calculated for all demographic variables. The two original questionnaires—the Patient Appraisal of the DOTS Program Efficacy and the Nurse Assessment of the DOTS Program Practice—were analyzed using item analysis (inter-item correlation coefficients) and exploratory factor analysis (Promax rotation) to examine and validate the questionnaires’ structures. Cronbach’s α was used to estimate their validity. The correlation analysis between the PTGI-SF scores (0–50) and each dependent variable was conducted using Pearson’s correlation coefficient. The PTGI-SF scores were determined to be normally distributed using a normality test (*p* = 0.01). Therefore, participants were assigned to a “high post-traumatic growth” (1) or “low post-traumatic growth” (0) group with reference to the means in the analysis. All variables that were significantly correlated (*p* < 0.05) with the PTGI-SF scores (0–50) were adopted as explanatory variables for the PTGI-SF scores (high = 1/low = 0) in a logistic regression analysis that used the forced entry method, with consideration of the multicollinearity between the independent variables. IBM SPSS Statistics for Windows v. 22.0 software (IBM Corp., Armonk, NY, USA) was used, with the significance level set at 5%.

### Qualitative analysis

Verbatim transcripts of the open-ended items were prepared in the thematic analysis. Responses to the first question were used to explore treatment barriers, responses to the second question were used to explore treatment drivers, and responses to the third question were used to explore the outcomes of the DOTS program with respect to medication adherence, lifestyle, and mindset. First, the responses to the three questions were split into sentence chunks, with the smallest unit codified before categorizing the responses further using similar codes. Through the collection of the categorized codes, common significant content was identified and extracted. This repeated task of code/sub-category extraction allowed for the exploration and comparison of multiple sub-categories in order to name the categories and observe their similarities and differences. Regarding the categories of the treatment barriers, treatment drivers, and outcomes of the DOTS program, the task was repeated by advancing or regressing sequentially while considering the kinds of factors being generated [18]. To increase the validity of the analysis, two separate meetings with 10 participating public health nurses were conducted. After explaining the categorization, we obtained their opinions as to whether the categories deviated greatly from their experiences as caregivers. Then, we strove to refine the categories through repeated modification.

### Ethical considerations

This study was conducted with the approval of the ethics review committee of St. Luke’s International University, with which the author was affiliated at the time of the study (approved February 2014; no. 13–065). In addition, if the patients consented to their attending nurse’s involvement in answering the treatment questions, the nurses identified the patients by their date of birth. Then, the patients who consented to these procedures wrote down their date of birth by themselves. Answering the questionnaire and writing their own date of birth (if they permitted the nurse’s involvement) indicated written consent to participate in the study. The entire process was clearly explained in the questionnaire, and permission was received from the ethics review committee.

## Results

### Patient demographics and clinical characteristics (Table 1)

Questionnaires were returned by 127 patients and 88 public health or clinical nurses. The analysis dataset consisted of responses from 125 participants after excluding two respondents who left numerous questions unanswered. When they were diagnosed with TB, 97 (77.6%) were living in their own homes, while 26 (20.8%) were homeless, living in common lodging houses, on the streets, incarcerated, or in a similar situation. In addition, 62 (49.6%) were living alone, while 61 (48.8%) were living with another person. Forty-eight participants (38.4%) were unemployed, 27 (21.6%) were in “precarious employment” (e.g., day labor, or temporary or dispatch work), and 26 (20.8%) were employed full-time. In terms of their educational backgrounds, 47 (37.6%) had graduated only from junior high school, 52 (41.6%) had received only high school education, and 23 (18.4%) had graduated from university or another higher education institution.

**Table 1:** Demographics and clinical characteristics of patients with TB in Japan

	Frequencies or mean (SD)	*N* = 125
		% or (range)
Age (years)	63.3 (15.8)	(22–90)
Gender		
Male	98	78.4
Education		
Junior high school	47	37.6
High school	52	41.6
University or higher	23	18.4
Residence at time of diagnosis		
Own residence	97	77.6
Homeless	26	20.8
Household at time of diagnosis		
Living alone	62	49.6
Living with family, others	61	48.8
Occupation at time of diagnosis		
Unemployed	48	38.4
Precarious employment	27	21.6
Full-time employment	26	20.8
Other	21	16.8
Ability to move at time of diagnosis		
Disabled	30	23.6
Slightly disabled	19	15.0
Able	74	58.3
Comorbid diseases (multiple answers)		
None	46	36.8
Cardiovascular disease	33	26.4
Diabetes	22	17.6
Mental illness	11	8.8
Cancer	10	8.0
Liver disease	10	8.0
Other	29	23.2
Symptom severity		
Mild	64	51.2
Moderate	34	27.2
Severe	27	21.6
Number of previous TB treatments		
0	110	88.0
1	12	9.6
≥2	3	2.4
DOTS hospitalization duration		
< 1 month	15	12.0
1–2 months	34	27.2
≥ 3 months	24	19.2
Hospitalized for another disease	4	3.2
Outpatient only	48	38.4
DOTS treatment duration		
< 6 months	13	10.4
6 months	39	31.2
7–11 months	27	21.6
> 12 months	10	8.0
Patient doesn’t know	23	18.4
DOTS consultation (multiple answers)		
Physician	96	76.8
Public health nurse	92	73.6
Clinical nurse	47	37.6
Family	30	24.0
Others	10	8.0
Understanding of TB and DOTS		
Full	98	78.4
Slight	19	15.2
Poor	7	5.6
*Appraisals of the efficacy of the DOTS program*	29.4 (7.0)	(5–36)

Many participants (79: 63.2%) had comorbid diseases at the time of their diagnosis; for most participants (88.0%), the DOTS program was their first TB treatment, while 15 (12.0%) had received treatment at least twice previously. Therapy lasted 6, 7–11, and ≥ 12 months for 39 (31.2%), 27 (21.6%), and 10 (8.0%) participants, respectively, and 13 (10.4%) completed their treatment course in under 6 months. Moreover, 23 participants (18.4%) did not know how long they had received treatment, having failed to receive an explanation from their doctor or nurse or simply forgotten. Forty-eight participants (38.4%) received only outpatient services. However, the majority had been hospitalized for their treatment: 15 (12.0%) for < 1 month, 34 (27.2%) for 1–2 months, and 24 (19.2%) for ≥3 months.

TB symptom severity was reported as mild, moderate, or severe by 64 (51.2%), 34 (27.2%), and 27 participants (21.6%), respectively. Ninety-eight participants (76.8%) claimed to understand their disease and treatment course after an explanation, while 19 (15.2%) reported “little understanding,” and 7 (5.6%) reported “no understanding.”

### Responses from the nurses about the characteristics of the DOTS program (Table 2)

The majority of attending nurses provided the DOTS program once or twice per month (*n* = 45: 51.1%). In addition, 15 (17.0%) and 21 (23.9%) nurses provided support once or twice per week and almost daily, respectively. Four (4.5%) nurses explained that they have no regular schedule. Thirty respondents (34.1%) had performed home visits for their patients, 30 (34.1%) had their patients come to public health centers for treatment, and 16 (18.2%) had provided the DOTS program over the phone or via email. Some nurses reported employing multiple DOTS program approaches.

**Table 2:** Community DOTS characteristics in Japan (nurse responses)

	Frequencies or mean (SD)	*N* = 88
		% or (range)
Community DOTS frequency (multiple answers)		
Everyday	21	23.9
Once or twice a week	15	17.0
Once or twice a month	45	51.1
At beginning of Community DOTS	4	4.5
Other	3	3.4
Community DOTS measures (multiple answers)		
Home visits	30	34.1
Outpatient care: public health centers	30	34.1
Outpatient care: medical clinics	16	18.2
Phone call	14	15.9
Other	8	9.1
Community DOTS impediments (multiple answers)		
Side effects	28	31.8
Complications	21	23.9
Loss of contact	16	18.2
Drug-resistant TB	10	11.4
Dysfunction in family	8	9.1
Other	25	28.4
*Assessments of the DOTS program practice*	29.5 (4.4)	(18–36)

The DOTS program nurses reported multiple barriers to effective implementation of the program. These included side effects (*n* = 28: 31.8%), complications (21: 23.9%), loss of contact (16: 18.2%), drug-resistant TB (10: 11.4%), and dysfunction in family (8: 9.1%). The nurses identified more than one barrier per patient in some cases.

### The DOTS program outcomes (Table 3)

Based on the MMAS-4 criteria described above, 94 participants (75.2%) were classified as adherent and 31 (24.8%) as non-adherent. Sixty-seven participants (53.1%) reported full or partial recovery after starting the DOTS program, 46 (37.3%) noticed no change in their condition, and 12 (9.5%) stated that they felt worse. In addition, participants had a mean (± standard deviation) PTGI-SF score of 21.7 ± 11.1 points (range: 0–50).

**Table 3:**
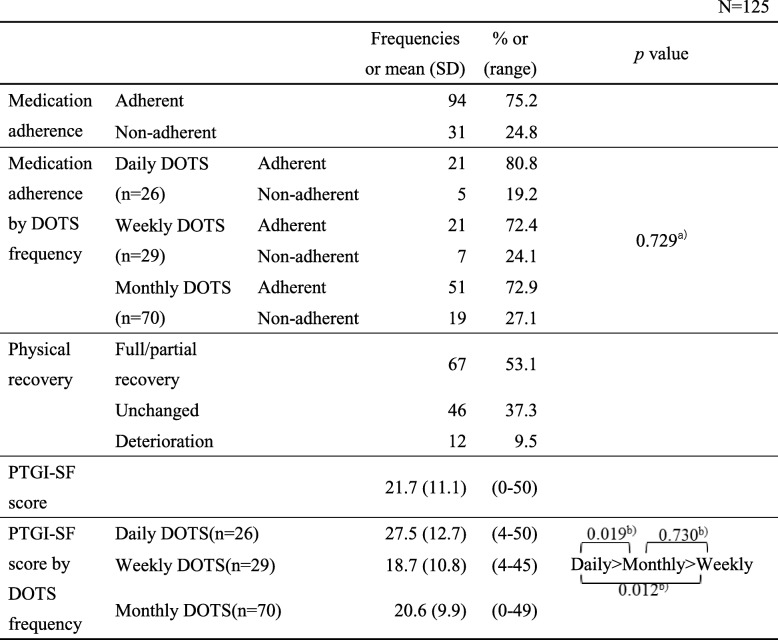
The DOTS outcomes in Japan

^a^ Chi-square test, ^b^ One-way analysis of variance with Tukey’s HSD

### Factors associated with post-traumatic growth (Tables 4 and 5)

The correlation analysis between the PTGI-SF scores (0–50) and eight independent variables were extracted as correlations (*p* < 0.05); all eight variables were adopted as explanatory variables for the PTGI-SF scores (high = 1/low = 0) in a binary logistic regression analysis using the forced entry method to test for associations between the PTGI-SF scores (between the high group and low group) and other factors in patients undergoing the DOTS program. Significant associations were observed for patients’ appraisals of the DOTS program efficacy (odds ratio [OR] = 1.157, 95% confidence interval [CI] = 1.026–1.304) and nurses’ assessments of the DOTS program practice (OR = 1.307, 95% CI = 1.065–1.603). In other words, participants exhibited greater post-traumatic growth when their recognition of the value of their DOTS program was high and the nurses’ assessments of the DOTS program protocols were more rigorous.

**Table 4:** Correlations for PTG in patients with TB receiving DOTS in Japan

	Correlation	*N* = 125	
		*p* value	
Age (years)	0.006	0.952	
Education	0.006	0.953	
Residence at time of diagnosis	−0.209	0.026	*
Household at time of diagnosis	−0.075	0.426	
Occupation at time of diagnosis	−0.058	0.545	
Ability to move at time of diagnosis	−0.197	0.037	*
Comorbid diseases at time of diagnosis	0.001	0.993	
Symptom severity	0.193	0.039	*
Number of previous TB treatments	0.152	0.105	
DOTS treatment duration	−0.064	0.520	
DOTS hospitalization	0.117	0.214	
Physician’s consultation	0.119	0.207	
Public health nurse’s consultation	0.054	0.567	
Clinical nurse’s consultation	0.198	0.033	*
Understanding of TB and DOTS	0.041	0.664	
DOTS frequency	0.209	0.024	*
*Appraisals of the efficacy of DOTS program*	0.247	0.010	*
*Assessments of the DOTS program practice*	0.300	0.007	**
Medication adherence	0.118	0.208	
Physical recovery	0.194	0.037	*

Pearson’s correlation coefficient, *: *p* < 0.05, **: *p* < 0.01

Education (0 = junior high school, 1 = high school, 2 = university or higher)

Residence at time of diagnosis (0 = homeless, 1 = own residence)

Household at time of diagnosis (0 = living alone, 1 = living with family, others)

Occupation (0 = unemployed, 1 = precarious employment, 2 = full-time employment)

Ability to move at time of diagnosis (0 = disabled, 1 = a little disabled, 2 = able)

Comorbid diseases (0 = none, 1 = comorbid)

Symptom severity (0 = mild, 1 = moderate, 2 = severe)

DOTS hospitalization (0 = none, 1 = experienced)

Physician’s consultation (0 = none, 1 = experienced)

Public health nurse’s consultation (0 = none, 1 = experienced)

Clinical nurse’s consultation (0 = none, 1 = experienced)

Understanding of TB and DOTS (0 = no understanding, 1 = a little understanding, 2 = understanding)

DOTS frequency (0 = monthly, 1 = weekly, 2 = everyday)

Medication adherence (0 = non adherence, 1 = adherence)

Physical recovery (0 = feel deterioration, 1 = unchanged, 2 = full/partial recovery)

**Table 5:** Factors associated with PTG in patients with TB receiving DOTS in Japan

	*N* = 88			
	95% CI			*p* value
	OR	Lower	Upper	
Residence				
Homeless	1.000			
Own residence	0.813	0.104	5.911	0.784
Symptom severity				
Mild	1.000			
Moderate	0.847	0.179	4.007	0.834
Severe	2.127	0.375	12.053	0.394
Physical recovery				
Feel deterioration	1.000			
Unchanged	1.764	0.200	15.568	0.609
Partial recovery	10.908	0.811	146.672	0.072
Full recovery	0.832	0.078	8.876	0.879
Clinical nurse’s consultation				
None	1.000			
Experienced	0.373	0.084	1.661	0.195
DOTS frequency				
Monthly	1.000			
Weekly	0.786	0.161	3.835	0.766
Daily	0.716	0.065	7.910	0.785
Ability to move at time of diagnosis				
Disabled	1.000			
A little disabled	0.288	0.040	2.043	0.213
Able	0.424	0.094	1.923	0.266
*Appraisals of the efficacy of the DOTS program*^a)^	1.157	1.026	1.304	0.017
*Assessments of the DOTS program practice*^b)^	1.307	1.065	1.603	0.010

Multivariate logistic regression analysis, forced entry method

*: *p* < 0.05

^a^Higher scores reflect greater efficacy

^b^Higher scores reflect greater practice

### Qualitative analysis of the open-ended responses (Table 6)

The participants’ open-ended responses to the question “What kind of things did you find troublesome as you went through the treatment?” were classified into the “treatment barriers” concept, while responses to “What kind of things did you find helpful as you went through the treatment?” were classified into the “treatment drivers” concept. The categories of “Non-acceptance,” “Frustration,” and “Anxiety” were extracted for the former, while “Fear,” “Acquiring a partner,” “Relief,” and “Belief” were extracted for the latter.

**Table 6:**
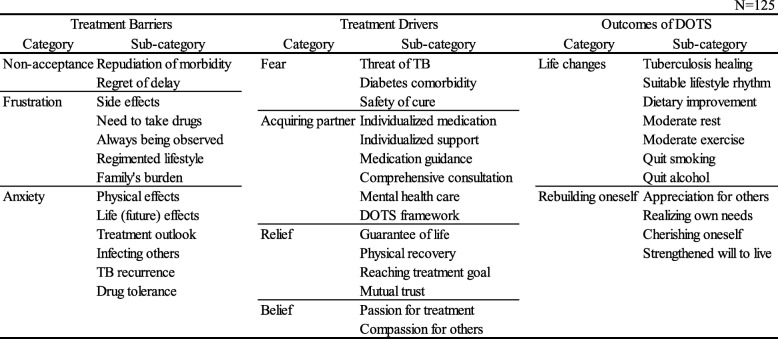
Experiences of patients with TB receiving DOTS in Japan

The participants’ open-ended responses to “How did your feelings, thoughts, behaviors, and lifestyle change after starting the treatment?” were classified as the DOTS program outcomes, from which two categories were extracted: “Life changes” and “Rebuilding oneself.”

## Discussion

Considering the patients’ psychological changes, qualitative research involving interviews with public health nurses who are responsible for providing medication and treatment support for patients with TB at high risk of discontinuation has shown that these programs not only improve treatment compliance but also help patients to develop healthy lifestyles [25]. Other studies have demonstrated the supportive environment for treatment continuation [26]. Support networks could be a useful source of information regarding TB patients’ perceptions of interventions, such as the DOTS program, in terms of their understanding of their disease and treatment course, which could be used to confirm their motivation for undergoing treatment.

In the current study, the participants’ mean PTGI-SF score was 21.7 ± 11.1 points. The DOTS program allows patients to move forward after a crisis state (i.e., being infected with a deadly disease) to positively affirm their own efforts to seek treatment, and to hear and internalize the encouraging voices of their supporters. These steps foster post-traumatic growth, which makes patients truly feel that their treatment has been effective, and they achieve a sense of accomplishment. The DOTS program provided patients with effective encouragement and motivation, which helped them to perceive its therapeutic effects and feel a sense of achievement. The existence of supporters is effective in helping individuals to achieve post-traumatic growth [27]. Support from others has previously been identified as an important factor associated with psychological growth [28]. Other authors have highlighted the importance of “cognitive, existential, humanistic, and narrative approaches” in driving growth, and of a “model of ‘expert companionship’ that focuses on the constancy of the companion, humility, respect for the survivor’s narrative and perspective, and a highlighting of strength” [27]. One of the treatment drivers that appeared as “Acquiring partner (Table 6)” coincided with “expert companionship,” suggesting that such professionals need to stay involved if patients are to experience post-traumatic growth. The inherent qualities of the DOTS programs—that is, comprehensive support customized to the patient’s characteristics—appear to be essential to the patients’ ability to move on from a major life crisis toward physical and psychological recovery and to rebuild their lives.

On the other hand, regarding the drug adherence that is the DOTS program outcome, in total, 75.2% of the study population was adherent (compliant with medication), which appears to be almost the same as the rates reported by other studies that used the MMAS-4 to evaluate adherence. For example, 65.4% of participants were considered adherent in China [29], 76.0% of participants were considered adherent in South Africa [30], and 75.3% of participants who attended TB clinics were considered adherent in Southern Ethiopia [31]. While the current study used the MMAS-4, healthcare professionals should exercise caution in issuing definitive judgments about a population’s adherence, as different tools can capture the concept in different ways, and its interpretation could be influenced by the nationality, community, and culture of the sample population. The observation of a 24.8% non-adherence rate in this study, which is the first to assess the medication status of patients with TB in Japan through direct inquiry, emphasizes the danger of unfounded optimism and suggests that continued efforts are necessary to support patients’ compliance with medication and treatment [32].

Regarding implications for practice, the DOTS program in Japan not only improved medication adherence rates but also increased levels of psychological growth. Reducing the TB prevalence by means of the Japanese version of the DOTS program is one of the most pressing challenges in public health, yet also one of the toughest to solve given the high concurrence of homelessness and alcohol and drug dependence in this population. The findings of the current study show that such high-risk patients with TB completed a journey in rebuilding their lives thanks to the DOTS programs, as they recognized their own issues and problems, gave thanks to others, and recovered their self-confidence. While direct observation, communication with patients, and guidance are crucial components of the DOTS program in Japan, the program strives to go beyond these components by supporting patients’ lifestyles with the help of family and supporters, customizing services to inspire them to improve their health habits, and standing by those who are enduring a great shock or stress [16]. The results of the current study constitute a reminder of the importance of the DOTS program as a comprehensive and continuous care program that sensitively accommodates the individual traits of every patient [33]. At-risk populations could be effectively treated by the DOTS-like programs that combine early detection with the provision of comprehensive, continuous, and individualized support.

The DOTS program in Japan is one example of a comprehensive community care system [34], which is a paradigm that involves systematic collaboration between various healthcare institutions, such as medical care facilities, public health centers, clinics, welfare facilities, and pharmacies. Effective collaboration in such a framework faces a variety of problems and challenges. In practice, care should be both individualized and smoothly implemented. Public health nurses face the largest burden of all, as they are required to motivate patients to improve their lifestyles, provide tailored support that is sensitive to the feelings of individual patients, and customize and develop a care framework that suits the needs and spirit of the times. Yet, Japan is well on its way to being classified as a low-TB incidence country, with rates dropping year after year, even as Japanese society increases in age. The role of the DOTS program in Japan in these efforts is essential, and the most salient contributing factor appears to be the customization of treatment frequency and support to suit the characteristics of target populations that involves a partnership.

## Limitations and future studies

The findings cannot be interpreted as denoting causal relationships with post-traumatic growth-related factors, because of the cross-sectional study design. Further, the study did not account for several conditions and experiences that could have inhibited post-traumatic growth. For example, some patients had TB pleurisy, while others had comorbid diseases, mental illness, or TB recurrence. Moreover, others were older adults who had lived through World War II. New data should be collected and analyzed while controlling for these variables. In addition, the low retrieval rate should be considered; it is believed that the sample size did not represent the population, which has been proven to be a significant limitation in the examination of PTGI-related factors. Further, the open-ended questions were not asked in a way that clearly allowed the patients to judge whether the DOTS program caused changes in their attitudes and daily activities. Therefore, it cannot be refuted that factors other than the DOTS program (during treatment) might have had some impact. Moreover, in this study, the public health nurses who were the patients’ attending nurses were asked to explain the study to the patients. Thus, it is likely that the patients were selected based on the judgments of these nurses. The patients to whom the explanations were given might have been in a psychological position that caused them to feel uncomfortable about refusing to participate in the study. This situation could have skewed the research results in a positive direction for the public health nurses. However, there is the possibility that the DOTS program, which places importance on the partnership between patients and care providers, contributes to the post-traumatic growth of a vulnerable population with a high discontinuation risk. This contribution provides a foothold for presenting the results both quantitatively and qualitatively. A valid and refined quantitative and qualitative evaluation (i.e., the Patient Appraisal of the DOTS Program Efficacy and the Nurse Assessment of the DOTS Program Practice) needs to be continued.

## Conclusions

The DOTS programs in Japan both improve treatment adherence in patients with TB and enhance their post-traumatic growth. The patients’ appraisals of the DOTS program’s efficacy and nurses’ confirmation of the DOTS program’s implementation may be important contributors to this positive psychological growth. Future work on the subject should endeavor to study the concept in greater detail and to select and refine the evaluation measures.

## Supplementary information

**Additional file 1: Table S1.** DOTS Program Efficacy—Items of Patient Appraisal.

**Additional file 2: Table S2.** DOTS Program Practice—Items of Nurse Assessment.

## Data Availability

The datasets generated and/or analyzed in the current study are not publicly available in order to protect the participants’ personal information, but they are available from the corresponding author on reasonable request.

## Abbreviations

CI: Confidence interval
DOTS: Directly observed treatment short-course
MMAS-4: Four-item Morisky Medical Adherence Scale
OR: Odds ratio
PTGI-SF: Post-Traumatic Growth Inventory-Short Form
TB: Tuberculosis
