# Supplementary material for: Psychological changes and associated factors among patients with tuberculosis who received directly observed treatment short-course in metropolitan areas of Japan: quantitative and qualitative perspectives
Source: BMC Public Health. 2019 Dec 5;19:1642. doi: 10.1186/s12889-019-8001-9 (PMC6896780; doi:10.1186/s12889-019-8001-9)
Supplement: Supplementary file 1 — Additional file 1: Table S1. DOTS Program Efficacy—Items of Patient Appraisal. [file 12889_2019_8001_MOESM1_ESM.docx]

Additional file 1: Table S1

DOTS Program Efficacy: Items of Patient Appraisal

Please circle the number that best matches your feelings about the support you have received from the staff involved in your treatment (at public health centers, hospitals, pharmacies, etc.)

|  |  | **Strongly agree** | **Agree** | **Neutral** | **Disagree** | **Strongly disagree** |
| --- | --- | --- | --- | --- | --- | --- |
| **1** | **Staff members understand the discomfort I periodically go through.** | **4** | **3** | **2** | **1** | **0** |
| **2** | **Staff members give a great deal of thought to me and my disease.** | **4** | **3** | **2** | **1** | **0** |
| **3** | **Staff members have explained aspects related to my disease and treatment plan clearly and correctly.** | **4** | **3** | **2** | **1** | **0** |
| **4** | **Staff members understand the effort I put into continuing my treatment.** | **4** | **3** | **2** | **1** | **0** |
| **5** | **Staff members support me by coordinating with hospitals, public health centers, pharmacies, and various individuals.** | **4** | **3** | **2** | **1** | **0** |
| **6** | **When my family or I are troubled, staff members take significant effort to resolve our issues.** | **4** | **3** | **2** | **1** | **0** |
| **7** | **When my family or I are troubled or conflicted, staff members are available to consult with about what to do.** | **4** | **3** | **2** | **1** | **0** |
| **8** | **Staff members have given me lifestyle-related advice.** | **4** | **3** | **2** | **1** | **0** |
| **9** | **Staff members will put forth their best effort through to the final day of my treatment.** | **4** | **3** | **2** | **1** | **0** |
