# Supplementary material for: Psychological changes and associated factors among patients with tuberculosis who received directly observed treatment short-course in metropolitan areas of Japan: quantitative and qualitative perspectives
Source: BMC Public Health. 2019 Dec 5;19:1642. doi: 10.1186/s12889-019-8001-9 (PMC6896780; doi:10.1186/s12889-019-8001-9)
Supplement: Supplementary file 2 — Additional file 2: Table S2. DOTS Program Practice—Items of Nurse Assessment. [file 12889_2019_8001_MOESM2_ESM.docx]

Additional file 2: Table S2

DOTS Program Practice: Items of Nurse Assessment

Please circle the number that best reflects your opinion about each of the following statements concerning your provision of DOTS to the patient in question.

|  |  | **Strongly agree** | **Agree** | **Neutral** | **Disagree** | **Strongly disagree** |
| --- | --- | --- | --- | --- | --- | --- |
| **1** | **I adjust my patient’s care contents (including initiating long-term care and home-visit nursing services) and make lifestyle recommendations to make it easier for them to continue treatment.** | **4** | **3** | **2** | **1** | **0** |
| **2** | **I give a great deal of thought to my patient’s illness (including their symptoms).** | **4** | **3** | **2** | **1** | **0** |
| **3** | **I display a sympathetic attitude toward my patient to help reduce their discomfort.** | **4** | **3** | **2** | **1** | **0** |
| **4** | **I give a good explanation to my patient about aspects of their disease and treatment plan.** | **4** | **3** | **2** | **1** | **0** |
| **5** | **I verbally encourage and recognize my patient’s efforts to continue treatment (e.g., “You’re doing great!”)** | **4** | **3** | **2** | **1** | **0** |
| **6** | **I reach out to my patient’s family and key persons to gain their understanding and cooperation.** | **4** | **3** | **2** | **1** | **0** |
| **7** | **I communicate and coordinate with medical and community institutions to ensure that my patient takes their medication.** | **4** | **3** | **2** | **1** | **0** |
| **8** | **I am available for consultations about my patient’s complaints and concerns.** | **4** | **3** | **2** | **1** | **0** |
| **9** | **I encourage my patient to improve aspects of their lifestyle (e.g., meals, life rhythm, rest).** | **4** | **3** | **2** | **1** | **0** |
